# The Effect of *Cutibacterium acnes* Infection on Nerve Penetration in the Annulus Fibrosus of Lumbar Intervertebral Discs via Suppressing Oxidative Stress

**DOI:** 10.1155/2022/9120674

**Published:** 2022-02-27

**Authors:** Zhi Shan, Xianjun Wang, Wentian Zong, Jie Li, Bingjie Zheng, Bao Huang, Xuyang Zhang, Jian Chen, Yue Huang

**Affiliations:** ^1^Department of Orthopaedics, Sir Run Run Shaw Hospital, School of Medicine, Zhejiang University, No. 3, Qingchun Road East, Hangzhou 310016, China; ^2^Department of Orthopaedics, Linhai Second Hospital, No. 198 Dubei Road, Linhai, Taizhou 317000, China; ^3^Department of Orthopaedics, Ningbo Medical Center Lihuili Hospital, No. 57 Xingning Rd, Ningbo 315040, China

## Abstract

Modic changes (MCs) and low back pain are highly correlated and an economic burden to the society. Previous studies have shown that *Cutibacterium acnes* (*C. acnes*) infection can lead to MCs. The purpose of this study was to clarify whether and how *C. acnes* contributes to oxidative stress and nerve growth that potentially leads to low back pain. Neurons from the hippocampus or dorsal root ganglion (DRG) of Sprague-Dawley (SD) rats were cocultured with annulus fibrosus cells (AFCs) with or without the presence of the *C. acnes* supernatant *in vitro*. Cell viability, neurite length, oxidative stress, and neuro-related gene expression were examined. Furthermore, samples from the patients with MCs and SD rat model of MCs were used to validate the nerve growth results. Neurons from both the hippocampus and DRG showed neurites when cocultured with AFCs in the environment with/without the *C. acnes* supernatant. The average neurite length was significantly longer when exposed to the *C. acnes* supernatant in the hippocampal neuron (217.1 ± 90.0 *μ*m versus 150.1 ± 68.1 *μ*m in the control group) and in the DRG neuron (229.1 ± 91.3 *μ*m versus 149.2 ± 64.8 *μ*m in the control group). Hippocampal neurons showed upregulated expression levels of NeuN, Map2, and Psd95, while upregulation was only seen in Tuj-1 in DRG neurons. Suppressed oxidative stress could be observed using axon growth symbols. Degenerated disc structures and abnormal bone remodelling were found in animal models and clinical samples of MCs, with astrocytes, microglia, and neurons in the disc. Therefore, *C. acnes* infection was found to cause back pain in the presence of MCs by promoting nerve penetration into the annulus fibrosus by suppressing oxidative stress.

## 1. Introduction

Intervertebral disc degeneration (IDD) is the main source of low back pain, which causes over 80% of disability worldwide [1] and imposes substantial social and economic burdens [2]. The main pathological changes occurring in IDD include proteolytic degradation of the extracellular matrix (ECM) [3], disruption of the physical structure of the annulus fibrosus and endplate [4], and inflammatory cytokine accumulation within/around the disc [5]. IDD can be observed with multiple signs on magnetic resonance images (MRI), including disc height loss, nucleus pulposus signal intensity decrease in T2, high-intensity zone, and Modic changes (MCs). Among these, MCs have been confirmed to correlate with low back pain [6], though the aetiopathogenesis of MCs remains unclear. First described in 1988, MCs represent signal changes in the vertebral endplate and subchondral bone region visualised on T1- and T2-weighted magnetic resonance (MR) images and are classified into three types: increased signal intensity on T2-weighted images and decreased signal intensity on T1-weighted images as type I, increased signal intensity on both T2- and T1-weighted images as type II, and decreased signal intensity on both T2- and T1-weighted images as type III [7].

In the 1930s, free nerve fibre distribution was observed in normal lumbar intervertebral discs [8]. Fagan et al. [9] quantitatively analysed innervation with PGP 9.5 immunofluorescence and found that cartilage endplate innervation was concentrated centrally adjoining the nucleus. The anterior superficial annulus fibrosus was mostly innervated, but the nerves only penetrated the annulus fibrosus to a maximum thickness of seven lamellae. Multiple studies have demonstrated that infiltration of nerve fibres into the inner annulus fibrosus is the main neural mechanism of discogenic back pain [10–12]. In degenerated discs, nerve endings may infiltrate deeper into the inner area [10], and deeply innervated discs generate more pain [11]. Peng et al. [12] reported that when annular fissures occurred in degenerated discs, nerve fibres can grow along granulation tissue in the fissures and can penetrate into the nucleus pulposus. Recently, using gene-knockout mice to simulate intervertebral disc degeneration, Miyagi et al. [13] discovered nociceptive nerve fibres infiltrating discs. Although it is generally believed that innervation relies on vessel ingrowth, Binch et al. [14] demonstrated that nerves could be present within nucleus samples, even in the absence of blood vessels.

Oxidative stress is generally understood to be the result of unbalanced antioxidant and prooxidant metabolic process [15]. Oxidative stress can affect microtubule stabilisation and inhibit axonal growth [16], and by regulating oxidative stress, axonal regeneration can be improved after peripheral nerve injury [17]. In intervertebral discs (IVDs), oxidative stress is considered a risk factor leading to disc degeneration [18, 19], and it can be used as a target to retard disc degeneration [19].

*Cutibacterium acnes* (*C. acnes*), formerly known as *Propionibacterium acne*, is one of the most abundant bacteria on human skin and can cause many types of diseases when ectopic infection occurs [20, 21]. Infection of the IVD by *C. acnes* is associated with discogenic pain, and fifty-three percent of patients with lumbar disc herniation (LDH) have anaerobic microorganism (*C. acnes* or *Corynebacterium propinquum*) infection [22]. Albert et al. showed that endplate MCs were related to the presence of *C. acnes* [23], and treatment with antibiotics (amoxicillin/clavulanate) can achieve better clinical pain relief [24]. Dudli et al. [25] induced MC type I with *C. acnes* stimulation in an ex vivo rat model, and Shan et al. [26] further established MC type I- and II-like changes in an *in vivo* rabbit model. Recent evidence suggests that exposure of primary human IVD cells to *C. acnes* significantly upregulates the expression of inflammatory cytokines [27]. However, there is a lack of research on *C. acnes* and nerve ingrowth in IVD, and the role of oxidative stress is unclear.

In the present study, we investigated the role of *C. acnes* and its contribution to nerve growth in *in vitro* and *in vivo* methods using clinical samples and Sprague-Dawley (SD) rat model.

## 2. Materials and Methods

All protocols in this study were approved by the Ethics Committee of Sir Run Run Shaw Hospital, Zhejiang University, Hangzhou, in compliance with the Guide for the Care and Use of Laboratory Animals published by the US National Institutes of Health (NIH publication no. 85-23, revised 1996).

### 2.1. In Vitro Cell Coculture of AFCs and Neuron

#### 2.1.1. Hippocampal Neuron Preparation

Primary embryonic hippocampal neuron cultures were prepared as previously described [28]. The hippocampus was harvested from SD rats (E18), digested with 1% collagenase II (Sigma-Aldrich, San Francisco, CA) at 37°C for 3 hours, and centrifuged (1500 rpm, 5 minutes); then, the hippocampal neurons were cultured on the coverslips coated with 100 *μ*g/ml poly-D-lysine. The culture medium consisted of Neurobasal, N2 (100x), B27 (50x), GlutaMAX-I (100x), and streptomycin-penicillin (100x). The medium was half replaced every three days.

#### 2.1.2. DRG Neuron Preparation

Lumbar DRGs were harvested from SD rats of 8 weeks immediately after sacrifice. The DRGs were rapidly dissected from the nerve roots, minced in PBS, and digested with 1% collagenase II (Sigma-Aldrich, San Francisco, CA) at 37°C for 3 hours before centrifugation (1500 rpm, 5 minutes). DRG neurons were then cultured on the coverslips coated with 100 *μ*g/ml poly-D-lysine, with the culture medium consisting of Neurobasal (Gibco, CA), N2 (100x, Gibco, CA), B27 (50x, Gibco, CA), GlutaMAX-I (100x, Gibco, CA), and streptomycin-penicillin (100x). The medium was half replaced every three days.

#### 2.1.3. AFC Preparation

Primary AF cells were harvested from 8-week-old SD rats. After sacrificing the SD rats with carbon dioxide, the IVD were isolated, and the outer AF were sharply dissected and enzymatically digested with 1% collagenase II (Sigma-Aldrich, San Francisco, CA) for 2 hours at 37°C in a 5% CO_2_ incubator. Then, the isolated AF cells were collected and cultured in DMEM (Gibco, CA) containing 10% FBS (Gibco, CA) with 1% penicillin/streptomycin under 5% CO_2_ at 37°C. Every other day, the culture medium was changed and the cells were harvested and/or passaged when confluent. Cells within passage 4 were used for further study.

#### 2.1.4. *C. acnes* Supernatant Preparation

*C. acnes* (1 × 10^7^ CFU/ml, ATCC 6919, Guangdong, China) were cultured with TSB in anaerobic environment at 37°C for 2 weeks. The bacteria were then centrifuged at 4000g for 5 minutes, suspended in PBS with the same concentration of 1 × 10^7^ CFU/ml, cultured in an anaerobic bag (AnaeroPack, Mitsubishi, Japan) at 37° for 2 days, and centrifuged again at 4000g for 20 minutes. After being filtered with a 220 nm molecular sieve twice, the supernatant was collected for further experiment as 1x concentration.

#### 2.1.5. Coculture of AFCs and Neuron, with/without the Presence of the *C. acnes* Supernatant

After being cultured on the coverslips for 7 days, the neurons were added with AFCs (12,500 cells/cm^2^) and/or *C. acnes* supernatant (10x) and cocultured with the same medium under 5% CO_2_ at 37°C. In this section, the *C. acnes* supernatant was used for treatment instead of *C. acnes* to avoid cell infection. The coculture process lasted for 3 days; then, the cells were collected for the RT-qPCR study or fixed with paraformaldehyde (4%) for immunofluorescence staining and neurite length measurement.

### 2.2. Clinical Samples

**Figure 1:**
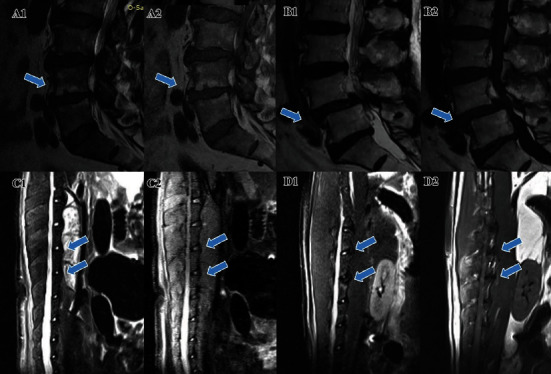
Representative MR image of the MC group (A1: T2-weighted; A2: T1-weighted) and control group (B1: T2-weighted; B2: T1-weighted). Representative preoperative (C1: T2-weighted; C2: T1-weighted) and postoperative (D1: T2-weighted; D2: T1-weighted) MR image of the MC animal model. Arrows indicate the segments with MCs or *C. acnes* injection. MCs: Modic changes.

Institutional review board approval and informed consent were obtained. From May to October 2017, ten consecutive patients with type I MCs who suffer from primary lumbar IDD were included (Figure 1), while another ten patients without MCs were consecutively included as the control. Patients with active infection or long-term infection history were excluded. Antibiotics were not applied for at least 3 months, except for a preoperative prophylactic cefuroxime dose of 1500 mg. All patients underwent lumbar magnetic resonance imaging (MRI), and a clinical diagnosis of IDD was confirmed according to the Pfirrmann grade (≥grade 3). All patients underwent transforaminal lumbar interbody fusion. The disc tissues, including the nucleus pulposus, annulus fibrosus, and cartilage endplate, were removed from the intradiscal space with a Kerrison Rongeur and curette, collected, and preserved with 4% paraformaldehyde immediately for hematoxylin-eosin (HE) staining and immunofluorescence staining later.

### 2.3. MC Model of SD Rats

All animal studies in the current research adhered to the ARRIVE checklist. Ten male SD rats (SLAC Laboratory Animal Co. Ltd., Shanghai, China) aged 8 weeks, weighing 150-180 g, were used to construct an MC model in their L3–4 and L4–5 segments according to the method previously reported [29], and the L2-3 and L5-6 segments were used as the shame and blank self-control. A small sample size is used this time because the model has been confirmed effective in our previous study [26]. Rats were anesthetized with intraperitoneal injection of pentobarbital sodium (15 mg/kg) and then laterally positioned on the platform. The L3–4 and L4–5 discs of all rats were injected with 10 *μ*l *C. acnes* (ATCC 6919 provided by Guangzhou Type Culture Collection (1.6 × 10^7^ CFU/ml supported with normal saline); the dose was referred from a previous animal model by Shan et al. [29]). The targeted IVD was exposed using a lateral retroperitoneal approach with aseptic techniques, punctured with a 32-gauge needle, and injected with the *C. acnes* solution. The incision was closed layer by layer; then, the animals were housed individually with free access to food and water. All the rats were monitored twice daily. Health was monitored by weight (twice weekly), food and water intake, and general assessment of animal activity, panting, and fur condition. Three months postoperatively, the rats were examined with MRI to identify MCs for each segment and then sacrificed with carbon dioxide. Spines with the targeted IVDs were harvested and dissected into several “vertebra-disc-vertebra” specimens and fixed with paraformaldehyde for further analysis. The surgery, MRI scanning, and sacrifice time were between 1700 pm and 2230 pm, and the testing order was randomized daily, with each animal tested at a different time each test day. The researchers who read the images and performed immunofluorescence were blinded for each observed disc.

### 2.4. Histology Sample Preparation

Samples from clinic and SD rats were fixed with 4% paraformaldehyde at 4°C for 24 hours and then decalcified at 25°C with 70% ethanol and decalcifying agent for 10 days. After being embedded in paraffin, samples were sectioned into 5 *μ*m slices for HE staining and immunofluorescence staining for NeuN, Tuj-1, Iba-1, and GFAP.

### 2.5. Immunofluorescence Staining

The coverslips or slices were postfixed with 4% paraformaldehyde for 24 hours at 4°C, washed three times with PBS, blocked for 2 hours with 0.1% Triton X-100 and 1% BSA at 25°C, and subjected to antigen repair with sodium citrate at 98°C for 20 minutes. The samples were incubated with primary antibodies NeuN (1 : 500, ET1602-12, HuaBio, Hangzhou, China), Tuj-1 (1 : 500, M0805-8, HuaBio, Hangzhou, China), Iba-1 (1 : 500, RT1316, HuaBio, Hangzhou, China), and GFAP (1 : 500, EM140707, HuaBio, Hangzhou, China) overnight at 4°C, washed three times with PBS, and incubated with secondary antibodies (1 : 500, A21121, Invitrogen, USA) for 2 hours at room temperature. For each clinical or animal sample, all immunofluorescence-positive cells from 3 random inconsecutive slices were picked.

### 2.6. Neurite Length Measurement

Neurite length measurement was performed with the NeuronJ plug-in based on ImageJ (National Institutes of Health, USA), in which the nerve fibers were identified as Tuj-1 (HuaBio, Hangzhou, China) positive in nerve fiber and NeuN (HuaBio, Hangzhou, China) positive in the cell body simultaneously. For each sample, measurement of the neurite length of all fibers was performed and calculated in 5 random 20x views, and three duplicate samples were investigated.

### 2.7. Oxidative Stress Measurement

The oxidative stress level was determined by MitoSOX™ staining (Yeasen, Shanghai, China), which is a specific red mitochondrial superoxide indicator in live cells. AFCs and DRG neurons for the test were incubated alone or cocultured for 1 day; then, the culture medium was removed and incubated with 5 *μ*M MitoSOX-Hanks buffer solution at 37°C in a 5% CO_2_ atmosphere protected from light for 30 min. Then, the cells were fixed with 4% paraformaldehyde for fluorescence observation. The N-acetyl-L-cysteine (NAC, a typical antioxidant) was used as a positive control of antioxidant at a concentration of 4 mM.

### 2.8. RT-qPCR

The total RNAs of cells from respective groups were extracted and purified by TRIZOL (Invitrogen) according to the manufacturer's protocols. After quality measurement with NanoDrop 2000, RNAs were reverse transcribed with PrimeScript RT Master Mix (Takara Bio, Otsu, Japan). RT-qPCR was performed using the SYBR Premix Ex Taq™ Kit (Takara, Dalian, China), and the primers (synthesized by Tsingke Biotech, Shanghai, China) used are listed in Table 1. The RT-qPCR cycling program was as follows: 95°C for 2 min, 40 cycles of 95°C for 10 sec, 60°C for 20 sec, and 72°C for 20 sec. The amplification signals from target genes were normalized by the glyceraldehyde-3-phosphate dehydrogenase (GAPDH) in the same reaction. Expression levels of the genes were determined using the relative quantification method.

### 2.9. Statistical Analysis

All quantitative data were presented as mean ± standard deviation. The *t*-test was used to compare mean data between two groups. The chi-square test was used to compare proportions. Analysis of variance (ANOVA) was used to compare mean data among groups, with differences between each group further analyzed with the least significant difference (LSD). The clinical sample size was calculated by using G∗Power 3.1.9.2 (University Kiel, Kiel, Germany). All statistical analyses were performed with SPSS 19.0 (IBM Corporation, New York, NY, USA), and a *P* value of <0.05 was considered significant.

## 3. Results

### 3.1. Coculture of Hippocampal Neuron and AFC Induces Neurite Growth, Which Is Further Enhanced by the *C. acnes* Supernatant

**Figure 2:**
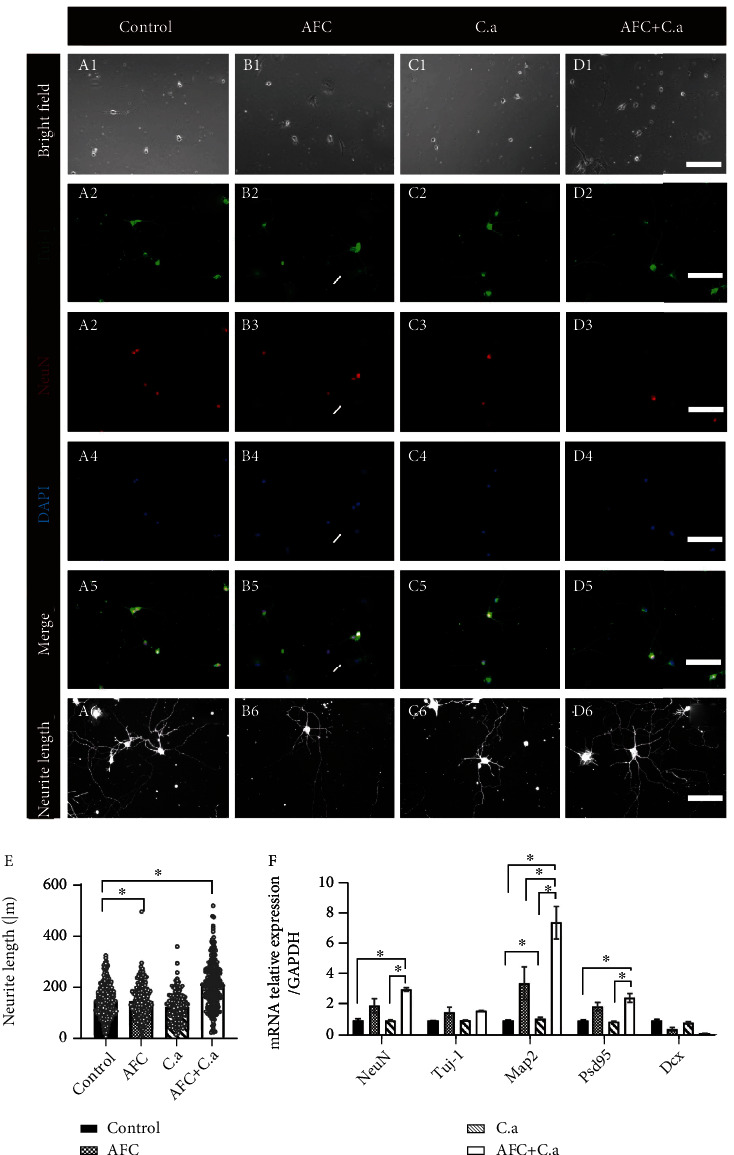
Representative images of cocultured AFCs and hippocampal neuron in bright-field, Tuj-1/NeuN/DAPI/merged immunofluorescence, and neurite length measurement (20x, A–D, scale bar = 100 *μ*m, the white arrows indicate where the neurite formed between neurons and AFCs). (e) shows the quantitative measurement of neurite length (means ± SD, ^∗^*P* < 0.0.5). (f) Nerve-related gene expression in hippocampal neuron-AFC coculture environment (means ± SD, ∗ indicates *P* < 0.0.5). AFCs: annulus fibrosus cells; C. a: *Cutibacterium acnes*.

The neurite growth ability of hippocampal cells when cocultured with AFC with or without the *C. acnes* supernatant is presented in Figure 2 (A–D). In all groups, no significant signs of cell degeneration were observed (Figure 2 A1–D1). In the control group, all cells were stained with NeuN (Figure 2 A3–A5) and clearly demarcated neurons, and most of these cells showed Tuj-1-positive neurites of various lengths (Figure 2 A2). In the AFC/hippocampal neuron coculture group, both AFCs (DAPI positive, NeuN negative) and hippocampal neuron (DAPI and NeuN positive, Figure 2 B3–B5) were observed. Almost all the neurons exhibited Tuj-1-positive neurites, and some of the neurites formed synapses with the AFCs (Figure 2 B2). In hippocampal neurons treated with the *C. acnes* supernatant, all cells were stained with NeuN as a control group (Figure 2 C3–C5), and most cells showed neurite outgrowth (Figure 2 C2). When cocultured cells were treated with the *C. acnes* supernatant, the NeuN^+^ neurons formed more neurites and more synapses with the NeuN^−^ AFCs (Figure 2 D2–D5). The neuron count of each group was similar, while the average length of growing neurites was 217.1 ± 90.0 *μ*m in the cocultured group treated with the *C. acnes* supernatant, significantly longer than those in the simple coculture group (150.1 ± 68.1 *μ*m), the *C. acnes* supernatant treatment group (127.6 ± 57.1 *μ*m), and the control group (152.4 ± 63.7 *μ*m) (Figure 2(e)). The RT-qPCR results were consistent with the immunofluorescence results. When cultured with AFCs, markers of mature neurites, Tuj-1 and Map2, and marker of axon growth, postsynaptic density protein 95 (Psd95), were significantly upregulated, and the expression of the mature neuron marker NeuN was also elevated. Map2 expression was further elevated when the supernatant of *C. acnes* was added. In contrast, the expression level of Dcx, an early neuronal differentiation indicator, was decreased when AFCs were introduced (Figure 2(f)). Therefore, we can conclude that AFCs can increase the neurite growth ability of hippocampal neurons, which can be further enhanced by the *C. acnes* supernatant.

### 3.2. Coculture with AFC and *C. acnes* Supernatant Led to Increased Neurite Growth in DRG Neuron

**Figure 3:**
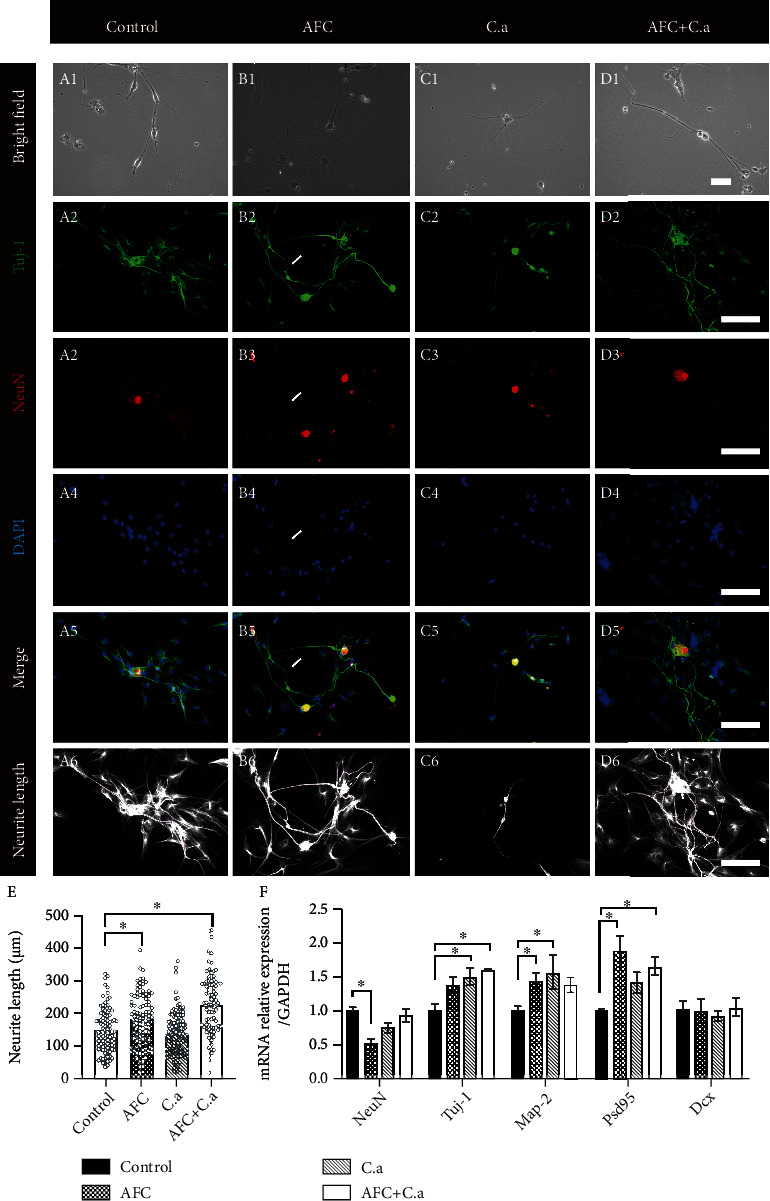
Representative images of cocultured annulus fibrosus cells and DRG neuron in (A) bright-field, Tuj-1/NeuN/DAPI/merged immunofluorescence, and neurite length measurement (20x, A–D, scale bar = 100 *μ*m; the white arrows indicate where the neurite formed among neurons and neurogliocytes or AFCs). (e) shows the quantitative measurement of neurite length (means ± SD, ^∗^*P* < 0.0.5). (f) Nerve-related gene expression in DRG neuron-AFC coculture environment (means ± SD, ∗ indicates *P* < 0.0.5). DRG: dorsal root ganglions; AFC: annulus fibrosus cells; C. a: *Cutibacterium acnes*.

No significant signs of cell degeneration were found (Figure 3 A–D). NeuN^+^ DRG neurons and DAPI-only neurogliocytes were observed in the control group. Most neurons showed Tuj-1 positive neurites of various lengths (Figure 3 A2). In the AFC/DRG neuron coculture group, more Tuj-1-positive neurites were found (Figure 3 B3–B5) and synapses between neurons and neurogliocytes or AFCs were observed (Figure 3 B2). DRG neurons treated with the *C. acnes* supernatant showed similar NeuN^+^ neurons and Tuj-1^+^ neurites compared to the control group (Figure 3 C2–C5). When treated with the *C. acnes* supernatant, cocultured AFC/DRG neurons showed increased neurite growth and synapse-forming ability (Figure 3 D3–D5). Neurite count and length measurements showed a significant increase in neurite length in the coculture group treated with the *C. acnes* supernatant (229.1 ± 91.3 *μ*m), followed by the DRG/AFC cocultured group (181.1 ± 79.1 *μ*m). The *C. acnes* supernatant exposure group (137.8 ± 63.0 *μ*m) and control group (149.2 ± 64.8 *μ*m) presented shorter neurite length, with no significant difference between them (Figure 3(e)). RT-qPCR showed that when cultured with AFC, the expression of neurite-associated markers Tuj-1, map2, and Psd95 was upregulated. In contrast, the expression of NeuN was decreased. The addition of the supernatant of *C. acnes* did not significantly upregulate the expression of Map2 and Psd95 but further upregulated the expression level of Tuj-1 slightly and elevated the expression of NeuN back to the level of the control group (Figure 3(f)). Unlike in hippocampal neurons, the expression level of Dcx remained unchanged in all groups. All results suggest that AFCs together with the *C. acnes* supernatant induced increased neurite growth ability of DRGs, although the pattern was slightly different from that of the hippocampus.

### 3.3. *Cutibacterium acnes* Supernatant Treatment Suppressed Oxidative Stress Level

**Figure 4:**
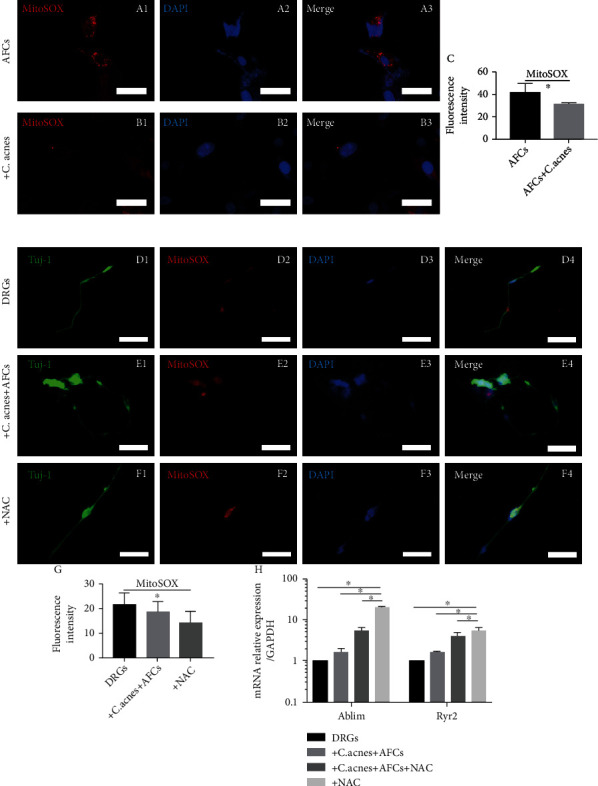
(A, B) Representative images of annulus fibrosus cells treated with/without the *C. acnes* supernatant, stained with MitoSOX and DAPI (40x, scale bar = 50 *μ*m), with MitoSOX density quantified in (c). (D–F) Representative images of DRG neurons cocultured with annulus fibrosus cells and *C. acnes* supernatant (40x, scale bar = 50 *μ*m), with MitoSOX density quantified in (g); (h) shows the Ablim and Ryr2 gene expression in DRG neuron-AFC coculture environment (means ± SD, ∗ indicates *P* < 0.0.5). AFCs: annulus fibrosus cells; C. a: *Cutibacterium acnes*; DRG: dorsal root ganglions.

MitoSOX staining showed suppressed oxidative stress in AFCs when incubated with the *C. acnes* supernatant (Figure 4 A–(c)) and in cocultured AFC/DRG neurons with the *C. acnes* supernatant (Figure 4 D–(g)). RT-qPCR revealed that the expression of Ablim (actin-binding LIM protein, a key axon guidance protein) [30] and Ryr2 (ryanodine receptor 2, regulating the intracellular calcium homeostasis and the establishment of neuronal polarity and axonal development, Figure 4(h)) [31] was upregulated when AFC/DRG were cocultured with the *C. acnes* supernatant and/or NAC. Both the *C. acnes* supernatant and NAC reduced the levels of MitoSOX and increased the nerve growth protein expression, suggesting that the *C. acnes* supernatant can induce nerve infiltration by suppressing oxidative stress.

### 3.4. Clinical IVD Samples with MCs Have More Nerve Ingrowth

**Figure 5:**
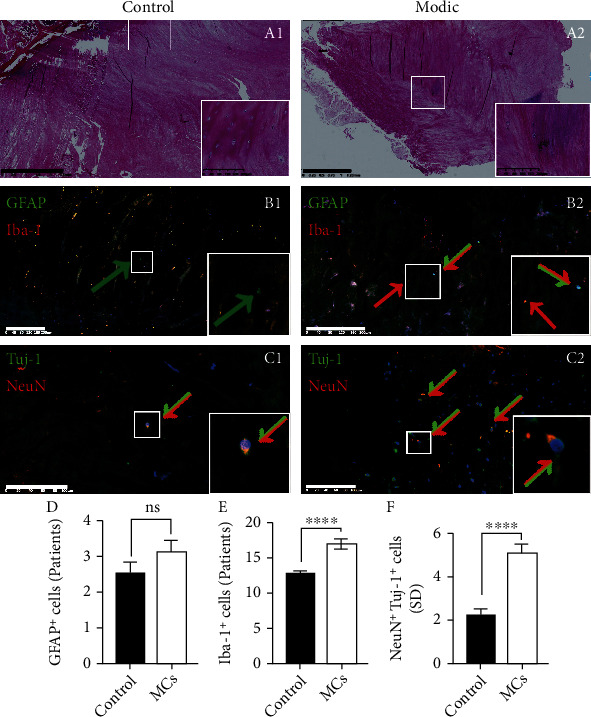
Representative images of the clinical MC group and control group with (A) HE staining (scale bar = 1.25 mm), (B) immunofluorescence of GFAP and Iba-1 (scale bar = 200 *μ*m), and (C) immunofluorescence of Tuj-1 and NeuN (scale bar = 200 *μ*m); images in the lower right corner are magnified from the boxed area of the corresponding images, with target protein indicated by coloured arrows. (d)–(f) show quantitative measurement of GFAP/Iba-1/NeuN-positive cells (means ± SD of a single slice, ∗ indicates *P* < 0.0.5). MCs: Modic changes.

The general information of patients in the MC and control groups showed no statistical difference (Table 2). HE staining of the junction between the annulus fibrosus, nucleus pulposus, and endplate demonstrated that samples from patients without MCs showed a clear boundary between the annulus fibrosus and endplate, while more haematoxylin-stained cartilage components within the annulus fibrosus region were observed in samples from the MC group (Figure 5A). Immunofluorescence examinations of identical samples showed GFAP^+^ astrocytes and Iba-1^+^ positive microglia in the IVD of the MC group, and the number of astrocytes and microglia in the control group was significantly lower (Figures 5B and 5(d) and 5(e)). In samples from patients without MCs, only a few neurons (stained with Tuj-1 and NeuN simultaneously) were stained, whereas a large number of neurons were observed in IVDs with MCs (Figures 5C and 5(f)). Taken together, these data demonstrate that cells from the neurosystem were more abundant in samples of degenerated discs with MCs.

### 3.5. MC Model of SD Rats Has More and Deeper Neuron Infiltration within IVDs

**Figure 6:**
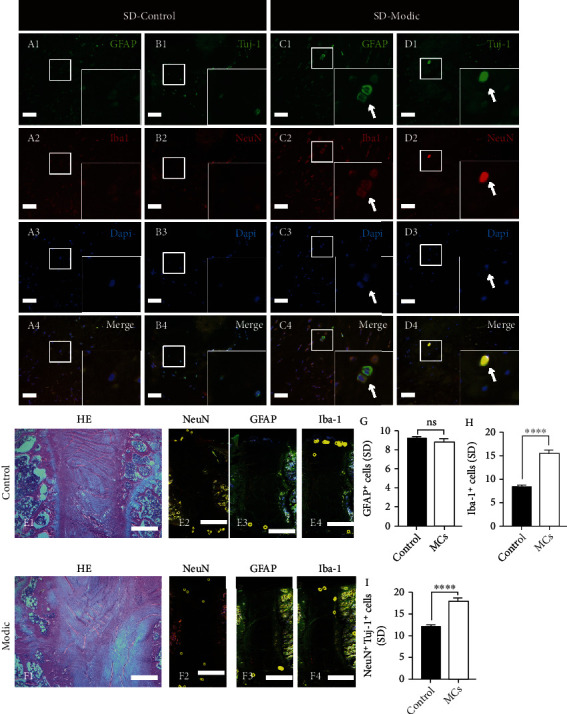
Representative images of the SD rat MC group and control group with immunofluorescence of GFAP/iba-1 (A and C, scale bar = 100 *μ*m) and Tuj-1/NeuN (B and D, scale bar = 100 *μ*m); images in the lower right corner are magnified from the boxed area of the corresponding images, and white arrows indicate the GFAP/Iba-1/NeuN-positive neurons. (E) and (F) show the location of GFAP/Iba-1/NeuN-positive neurons in the IVD (cells with positive immunofluorescence were marked as bright yellow dots (scale bar = 1 mm)); (g)–(i) show quantitative measurement of GFAP/Iba-1/NeuN-positive cells (means ± SD of a single motion segment, ∗ indicates *P* < 0.0.5). MCs: Modic changes.

Eleven of 20 segments treated with *C. acnes* were confirmed to have MCs by MRI three months postoperatively. No signs of disc degeneration or MCs were observed in the control segments. The assumptions of normality and homoscedasticity (standard deviations were equal) in our analyses were reasonable. At the microscopic level, immunofluorescence of Iba-1 indicated abundant microglial cells in the MC group, but much fewer in the control group. The GFAP^+^ positive astrocyte count was similar between the MC and control groups, while Tuj-1 and NeuN staining revealed a significantly larger cell count of neurons and neurites from samples of the MC group than from the control group (Figure 6A–D). At the macroscopic level, with HE staining, a degenerated disc structure and abnormal bone remodelling of the endplate area were seen in MC model segments, but not in control segments (Figure 6E–F). Immunostaining of GFAP and Iba-1 (performed with consecutive slices of the HE slices) indicated that astrocytes and microglia were located only in the peripheral area of the disc in the control group, whereas the cells grew deeper in the MC group. Immunofluorescence of NeuN demonstrated that some neurons penetrated the inner annulus fibrosus area in the MC group, but in the control group, they can only be seen at the edge of the annulus fibrosus (Figure 6E–F). Overall, the data suggested that the quantity and infiltration depth of cells from the neurosystem were higher in samples from the MC model.

## 4. Discussion

Our research examined how AFCs and MCs can affect nerve growth in IVDs. Using cell coculture, clinical samples, and animal models, we demonstrated that the annulus fibrosus could allow the neuralisation process and that this effect was enhanced when infection with *C. acnes* was apparent.

Physiologically, a healthy mature intervertebral disc is believed to be an avascular and noninnervated structure [32]. In the current study, both the hippocampus and DRG neurons showed neurite growth when cocultured with AFCs with/without the presence of the *C. acnes* supernatant. This is consistent with multiple previous studies that reported that MCs are correlated with innervation and low back pain [33, 34]. Several signs of degeneration of the annulus fibrosus, including disc bulge [35] and higher Pfirrmann grade [36], have been confirmed to coexist with MCs, and all signs are associated with painful symptoms in the back [6, 37]. Ohtori et al. examined inflammatory cytokines and nerve growth in the vertebral endplate and reported that vertebral endplates from patients with MC I or II had PGP 9.5-immunoreactive nerve fibres, but none in the control group, suggesting that endplate abnormalities are related to axon growth [33]. Miscusi et al. [34] examined clinical cases of degenerative disc disease with MCs and found that Nf200^+^ nerves in the MC I group were significantly higher than those in the MC II group (8.7% versus 2.3%), and more Nf200^+^ nerves were associated with higher disability scores.

In the hippocampus, the expression of DCX, an early neuronal differentiation marker, was downregulated when cocultured with AFCs and further downregulated by the supernatant of *C. acnes*, suggesting that neuralisation was achieved by neurite growth rather than neural cell proliferation and differentiation. Many previous studies have reported that neurogenesis can be induced by inflammatory factors, including IL-1*β* or nerve growth factor (NGF). In a recent study, Dudli et al. cocultured IVD cells and *C. acnes* and demonstrated an increase in IL-1, IL-6, and IL-8 mRNA expression in IVD cells [38]. With exogenous IL-1*β* treatment, Gruber et al. found significantly increased NGF production in AFCs, which is believed to induce neurogenesis and low back pain [39]. Using cell coculture, Gruber et al. [40] reported that IL-1-*β* or TNF-*α* significantly increased neurite length in F11 nerve cells. Oga et al. [41] reported that IL-1*β* mRNA expression was negatively correlated with the pressure pain threshold and might increase NGF levels in the skeletal muscle. Using anakinra, an IL-1R1-inhibiting antibody, Peng et al. successfully abolished IL-1*β*-induced neurogenesis *in vitro* [42], further confirming the ability of IL-1*β*. In our previous study, we also confirmed elevated expression levels of IL-1*β* in the IVD of an animal model of MCs induced by *C. acnes* [26].

Oxidative stress in the IVD can lead to disc degeneration and correlated symptoms [18, 19, 43, 44]. Surprisingly, we found that oxidative stress was suppressed by AFC coculture and *C. acnes* treatment but resulted in more neurite ingrowth. This seems contradictory to previous studies, which claim that *C. acnes* can increase oxidative stress. Lin et al. demonstrated that *C. acnes* upregulated the expression of reactive oxygen species (ROS) in NPCs via NADPH oxidase [43], while Tang et al. [44] reported that nucleus pulposus cells cocultured with *C. acnes* showed increased ROS in a time-dependent manner. However, reducing excessive oxidative stress may improve locomotor functional recovery after spinal cord injury [45]. Wang et al. [46] also confirmed axon regeneration after spinal cord injury by inhibiting oxidative stress. These contrasting results can be explained by the fact that excessive oxidative stress, leading to degeneration, mainly occurs in the central part of the IVD with insufficient blood supply. In the peripheral area (annulus fibrosus) with abundant blood supply, the suppression of appropriate oxidative stress by AFCs and *C. acnes* may result in abnormal ingrowth of nerve fibres. After treatment with NAC, an antioxidant, the current study showed elevated expression levels of Ablim and Ryr-2, which were similar to the results obtained when treated with AFCs and *C. acnes*, confirming that the nerve fibre-generating ability of AFCs and *C. acnes* was induced by suppressing ROS below physiological levels.

Compared to neuron from the hippocampus, cells treated with the supernatant of *C. acnes* induced more neurite growth in DRG. Interestingly, when the supernatant of *C. acnes* was added, RT-qPCR demonstrated that the expression of NeuN, map2, and psd-95 only tended to increase in hippocampal neurons but not in DRGs, which can be attributed to the fact that the DRGs already showed higher levels of expression of these genes at baseline, and the confounding effect of neurogliocytes in the DRG group diluted the effect of the *C. acnes* supernatant. This result was consistent with those of previous studies that reported different nerve development abilities in the DRG and hippocampus. According to Maday et al., distal enrichment of autophagosome biogenesis can be observed in both developing DRG neurons and hippocampal neurons, although the rates of distal formation in hippocampal neurons were lower than those observed in actively growing DRG neurons, suggesting higher dynamic states of growth cone extension and retraction in the DRG [47]. In another study, Auer et al. reported that RhoA plays a critical role in mediating the effects of myelin-derived inhibitors on axon outgrowth in the CNS; however, the functional significance of RhoA was limited in DRG [48]. Koch et al. [49] reported differential adaptation of cytoskeletal dynamics to culture substrate stiffness in growth cones of different neuronal types, which made DRG to have stronger substrate coupling in the cytoskeleton and higher traction force generation for nerve fibre growth in soft substrates. Furthermore, the DRG is much more mechanically sensitive than the CNS, which explains the relatively higher nerve-related gene expression of the DRG with AFCs in the current study. The extent of nerve-related gene upregulation in DRGs when introduced into the supernatant of *C. acnes* was smaller than that in the hippocampus, suggesting that more synapses can be induced in hippocampal neurons, while the DRGs have higher reactivity when cultured in Petri dishes, and neurons from either the hippocampus or DRGs showed similar neurite growth ability.

Our results and previous research revealed that infection of *C. acnes* in the disc elevated the neurite growth ability of neurons, while the impaired annulus fibrosus may provide the source for nerve fibre sprouting, leading to further annulus fibrosus damage and degenerative signs of the endplate, including MCs, nerve fibre and neuroglial ingrowth of the annulus fibrosus, and finally, discogenic pain (especially in the MC I phase). Thus, medicines targeting nerve fibre sprouting in patients with MCs may have a higher success rate of conservative management. Our results do not support antibiotic treatment for low back pain; *C. acnes* infection acts as an initiation of nerve growth, rather than a mechanism of pain. This also explains why antibiotic treatment seemed less useful in patients with existing MCs in the previous research [50].

One limitation of this study was that the *C. acnes* supernatant was used in the *in vitro* experiment. Although *C. acnes* is an aerotolerant anaerobic bacterium, it could not have been efficiently recovered in aerobic conditions, and its metabolic products in aerobic and anaerobic situation are different. However, nerve cells and annulus fibrosus cells must be cultured in aerobic conditions, and the slow recovery rate of *C. acnes* is not suitable for our experiment. Besides, both propionic acid and lipase, the most suspicious pathogen to dissolve bone and fatty bone marrow [38, 51], are soluble. Another limitation of our study was that only one MC model (induced by *C. acnes*) was evaluated, which is the only animal model for MCs ever reported. In addition, a needle puncture is a risk factor for disc degeneration and innervation. To reduce this negative effect, the needle size used was 32-gauge, which is much smaller than the size of the IVD and, therefore, much less damaging [52]. Furthermore, the sample size in the current study was relatively small. Studies on cellular and molecular mechanisms elucidating the signal transduction system of nerve ingrowth are needed in the future.

In conclusion, by suppressing oxidative stress and promoting nerve penetration into the annulus fibrosus, *C. acnes* infection can be the reason for low back pain in patients with MCs, and that can be a potential target for further treatment.

## Figures and Tables

**Table 1:** Nucleic acid sequence of forward and reverse RT-qPCR primers of specific genes.

Rat NeuN_F	5′-TATGCAGCTTACAGATATGCTC-3′
Rat NeuN_R	5′-CGCATAGACTCTACCATAACTG-3′
Rat Tuj_F	5′-TATCTTCGGTCAGAGTGGTG-3′
Rat Tuj_R	5′-CATCCAGGACTGAGTCCAC-3′
Rat Map2_F	5′-ACAGAGAAACAGCAGAGGA-3′
Rat Map2_R	5′-GTTCACCTTTCAGGACTGC-3′
Rat Psd-95_F	5′-AGATCCTGTCGGTCAATGG-3′
Rat Psd-95_R	5′-TCTGACCCGCATTCTTCAG-3′
Rat Dcx_F	5′-TGATGTGTTCATTGCTTGTG-3′
Rat Dcx_R	5′-ACTCTGCATTCATTCTCATCC-3′
Rat GAPDH_F	5′-ACAGCAACAGGGTGGTGGAC-3′
Rat GAPDH_R	5′-TTTGAGGGTGCAGCGAACTT-3′
Rat Ablim2_F	5′-GTGGCTTTGGACAAGCACTG-3′
Rat Ablim2_R	5′-TCTGCATTGAGGAGTTTCCCA-3′
Rat Ryr2_F	5′-GGGTGTCAGCGAAGGATCAG-3′
Rat Ryr2_R	5′-GGTCACAAAGGGTTCCGTGT-3′

**Table 2:** General information of clinical samples.

	MC group	Control group	*P* value
Gender			0.328
F	4	6	
M	6	4	
Age	62.70 ± 13.64	56.20 ± 15.70	0.336

## Data Availability

The datasets used and analyzed during the current study are available from the corresponding authors on reasonable request.
